# Serum Calcium Concentration Is Inversely Associated With Radiographic Knee Osteoarthritis: A Cross-Sectional Study

**DOI:** 10.1097/MD.0000000000002838

**Published:** 2016-02-12

**Authors:** Hui Li, Chao Zeng, Jie Wei, Tuo Yang, Shu-Guang Gao, Yu-Sheng Li, Wei Luo, Wen-Feng Xiao, Yi-Lin Xiong, Guang-Hua Lei

**Affiliations:** From the Department of Orthopaedics (HL, CZ, TY, S-GG, Y-SL, WL, W-FX, Y-LX, G-HL); Health Management Center (JW), Xiangya Hospital; and Department of Epidemiology and Health Statistics (JW), School of Public Health, Central South University, Changsha, Hunan Province, China.

## Abstract

To examine the relationship between serum calcium (Ca) concentration and radiographic knee osteoarthritis (OA).

This study covered a total of 2855 subjects. The serum Ca concentration was detected by the Arsenazo III method. The radiographic OA of the knee was defined as changes equivalent to Kellgren–Lawrence grade 2 on 1 side at least. The serum Ca concentration was categorized into 4 quartiles, which are ≤2.27, 2.28–2.34, 2.35–2.41, and ≥2.42 mmol/L, respectively. The relationship between serum Ca and radiographic knee OA was examined using the multivariable logistic analysis after adjusting a series of potential confounding factors. For each quartile of the relationship between serum Ca concentration and radiographic knee OA, the OR with 95% CI was calculated, and the one with the lowest value was considered to be the reference.

An inverse association existed between serum Ca concentration and radiographic OA of the knee in the multivariable model and the model where the factors of age, sex, and BMI were adjusted. The multivariable-adjusted OR (95% CI) for radiographic knee OA in the second, third, and fourth quartiles of serum Ca concentration were 1.05 (95% CI: 0.83–1.31), 1.01 (95% CI: 0.80–1.27), and 0.79 (95% CI: 0.62–1.00), respectively, in comparison with the reference (first) quartile. A trend approaching to statistical significant (*P* = 0.06) was observed. Meanwhile, the relative odds of radiographic OA of the knee were decreased by 0.79 times in the fourth quartile in comparison with the reference.

There is likely to be an inverse association between serum Ca concentration and radiographic OA of the knee.

## INTRODUCTION

Osteoarthritis (OA) is a degenerative disease of the joints which is characterized by degradation of articular cartilage, synovitis, and changes to subchondral bone which exhibits altered remodeling.^1^ It is a major public concern as it is one of the leading causes of morbidity and disability, thereby laying a huge medical and economic burden on health resources.^2^ Despite these concerns, the pathogenesis of OA remains unclear. A combination of local joint-specific factors acting in the context of systemic susceptibility may contribute to the development of OA. There seem to be many factors contributing to the occurrence and extent of the OA process including obesity, joint injury, metabolic diseases, bone and joint malformations, and genetic factors.^3^

It is hypothesized that nutritional imbalance is also involved in the pathogenesis of OA. The importance of nutrition in the maintenance of joint health is now widely recognized.^4^ In recent studies, our investigative group found that serum magnesium (Mg) concentration may have an inverse relationship with radiographic OA of the knee.^5^ Calcium, which belongs to the same family in the periodic table as Mg, shares the same homeostatic regulating system that involves calcium sensing receptor and (re)absorption with calcium.^6,7^ Meanwhile, Mg and calcium antagonize each other in many physiological activities.^7–10^ So the relationship of the prevalence of radiographic knee OA with serum calcium concentration is also worth studying. Calcium is an essential nutrient which plays a key role in regulating a great diversity of physiology processes, including muscle contraction, neurotransmitter release, endocrine and exocrine secretion, and blood clotting.^11,12^ In healthy individuals, serum levels of calcium are regulated by homeostatic mechanisms involving the calcium-sensing receptor, 1,25-dihydroxyvitamin D, and parathyroid hormone. Disorders of calcium homeostasis are related to an increased risk disease, such as cardiovascular disease, metabolic syndrome, and prostate cancer.^13–18^ There have been studies reporting associations between serum calcium and OA. However, no association was found between OA and serum calcium concentration in these studies.^19–21^ It is notable that these studies have been performed on Western population, the lifestyle and eating habits of which are different from Asian people. Differences may exist among populations from the different regions. For example, a multicenter hospital-based case-control study conducted in Asian revealed an inverse association between serum concentrations of calcium and the risk of prostate cancer,^18^ while no association was found in Western populations.^22,23^

Hence, thorough investigation of serum calcium concentration in Asian countries such as China can potentially reveal further insight concerning probable association between OA and serum calcium in a different geographic setting. We, therefore, designed a cross-sectional study aimed to further evaluate the association of the prevalence of radiographic knee OA with serum calcium concentration.

## METHODS

### Study Population

This cross-sectional study enrolled Chinese members of the general public who were undergoing health screening to explore the association between nutrition and the disease; the study design has been published previously.^5,24,25^ Such screening checkups have become routine in China, and generally include anthropometric and basic clinical assessment (e.g., weight, height, blood pressure and, waistline measurements, etc.), and biochemical (e.g., blood routine examination, hepatic function, renal function, trace elements test, etc.) and imaging (e.g., chest radiography and bilateral anteroposterior knee radiography, etc.) tests. Registered nurses collected details of demographic characteristics and health-related habits, such as age, occupation, educational level, physical activity level, smoking status, alcohol drinking status and, diet, using a standard questionnaire. Subjects were selected for this study according to the following inclusion criteria: age ≥40 years; availability of weight-bearing anteroposterior radiographs of both knees; availability of blood biochemistry including serum calcium and fasting glucose concentrations; availability of data on all basic characteristics, including age, sex, BMI, smoking status, etc. Initially, this cross-sectional study recruited 5486 participants who underwent routine checkups including weight-bearing bilateral anteroposterior radiography of the knee and blood biochemical tests, such as blood glucose, serum calcium concentration, at the Department of Health Examination Center of Xiangya Hospital, Central South University in Changsha, Hunan Province, China, from October 2013 to August 2014. Then, the following individuals were excluded: with radiographic evidence of non-OA joint disease, such as osteochondroma or fracture (n = 67), or with missing data on certain characteristics or physical examinations, such as BMI, waistline, and blood pressure (n = 2), or younger than 40 years (n = 539), or with missing data on the records of behavior habits, such as smoking status, alcohol using, and physical activity level (n = 2023). A total of 2855 subjects were included in the final analysis. This research was approved by the ethics committee of Xiangya Hospital of Central South University. All participants gave written informed consent at the time of recruitment.

### Blood Biochemistry

All blood samples were drawn after a 12-hour overnight fast and were stored at 4°C until analysis. The serum calcium concentration was measured using the Arsenazo III method. The fasting plasma glucose concentration was measured using the glucose oxidase enzyme method. Laboratory tests were undertaken using a Beckman Coulter AU 5800 (Beckman Coulter Inc, Brea, CA). Subjects with a fasting glucose ≥7.0 mmol/L or who were currently undergoing drug treatment for blood glucose control were regarded as having diabetes mellitus. The inter- and intra-assay coefficients of variation were tested by low concentrations (2.5 mmol/L for glucose, 118 μmol/L for uric acid, and 0.60 mmol/L for serum Mg) and high concentrations (6.7 mmol/L for glucose, 472 μmol/L for uric acid, and 1.00 mmol/L for serum Mg) of standard human samples. The intra-assay coefficients of variation were 0.98% (2.5 mmol/L) and 1.72% (6.7 mmol/L) for glucose, 1.39% (118 μmol/L) and 0.41% (472 μmol/L) for uric acid, and 1.86% (0.60 mmol/L) and 1.65% (1.00 mmol/L) for serum Mg. The interassay coefficients of variation were 2.45% (2.5 mmol/L) and 1.46% (6.7 mmol/L) for glucose, 1.40% (118 μmol/L) and 1.23% (472 μmol/L) for uric acid, and 1.87% (0.60 mmol/L) and 1.70% (1.00 mmol/L) for serum Mg.

### Assessment of Radiographic OA of the Knee

All subjects involved in this study received weight-bearing bilateral anteroposterior radiography of the knee. Two orthopedic surgeons examined all the radiographs independently according to the Kellgren–Lawrence (K–L) radiographic atlas without knowing the subjects’ clinical symptoms in advance. Disagreements between the 2 surgeons, if any, were resolved through discussion. OA was classified into 5 categories based on K–L grade: 0 = absence of OA; 1 = suspected OA; 2 = minimal OA; 3 = moderate OA; 4 = severe joint OA.^26^ Diagnosis of radiographic OA of the knee was confirmed if at least 1 knee joint was rated as K-L 2 or higher. Intraclass correlation coefficient (ICC) was used to judge the reliability of the 2 orthopedic surgeon’ assessments at 2 different times. The results indicated that both inter-rater and intra-rater reliability were high (κ=0.86 and 0.87, respectively).

### Assessment of Covariates

Blood pressure was measured using an electronic sphygmomanometer. Subjects with a systolic blood pressure ≥140 mm Hg or diastolic blood pressure ≥90 mm Hg, or who were currently using antihypertensive medication, were regarded as having arterial hypertension. BMI was calculated for each subject based on weight and height. All subjects were requested to provide information on average frequency of physical activity (never, once, or twice per week, 3 to 4 times per week, 5 times or above per week) and average duration of physical activity (30 minutes, 30–60 minutes, 60–120 minutes, >120 minutes), as well as the current smoking and alcohol drinking status (yes or no for each).

### Statistical Analysis

Continuous data are expressed as the mean ± standard deviation, and category data are expressed as proportion (percentage). The serum calcium concentration was classified into 4 quartiles: ≤2.27, 2.28–2.34, 2.35–2.41, and ≥2.42 mmol/L. Differences in continuous data were evaluated by the one-way classification ANOVA (Analysis of Variance, normally distributed data) or the Kruskal–Wallis H test (non-normally distributed data), whereas differences in category data were assessed by the χ^2^ test. Odds ratios (ORs) with 95% confidence intervals (CIs) for the association between radiographic knee OA and serum calcium concentration were calculated for each calcium concentration quartile; the quartile with the lowest value was regarded as the reference category. To calculate the adjusted OR of each quartile of serum calcium concentration, age, BMI, and sex were used as covariant at first. Then a multivariable model was adopted for logistic analysis that included age, BMI sex, educational level, smoking status, activity level, alcohol drinking status, diabetes, and hypertension. Tests for linear trends were conducted on the basis of logistic regression using a median variable of calcium concentration in each category. Subgroup analyses were conducted to assess the association between serum calcium concentration and radiographic knee OA in sex subgroup. All analyses were performed using SPSS 17.0; a *P* value ≤0.05 was considered statistically significant. All tests were 2-tailed.

## RESULT

The characteristics of the study population (2855 subjects) based on quartiles of serum calcium concentration are shown in Table 1. There were 1623 men and 1232 women, and the prevalence of radiographic OA of the knee in the present cross-sectional study (age ≥40 years, with an average age of 52.26 ± 7.16 years) was 30.0%. Serum calcium concentration of 570 subjects (20.0% of the study population) were below 2.25 mmol/L. Significant differences were observed across all quartiles of serum calcium concentration in terms of female ratio, alcohol drinking ratio, the ratio of diabetes, and the ratio of hypertension. There was no significant difference in terms of age (*P* = 0.12), sex (*P* = 0.14), or BMI (*P* = 0.96) between those included in the main analysis and those excluded as a result of missing health-related behavior data (n = 2023).

**TABLE 1:**
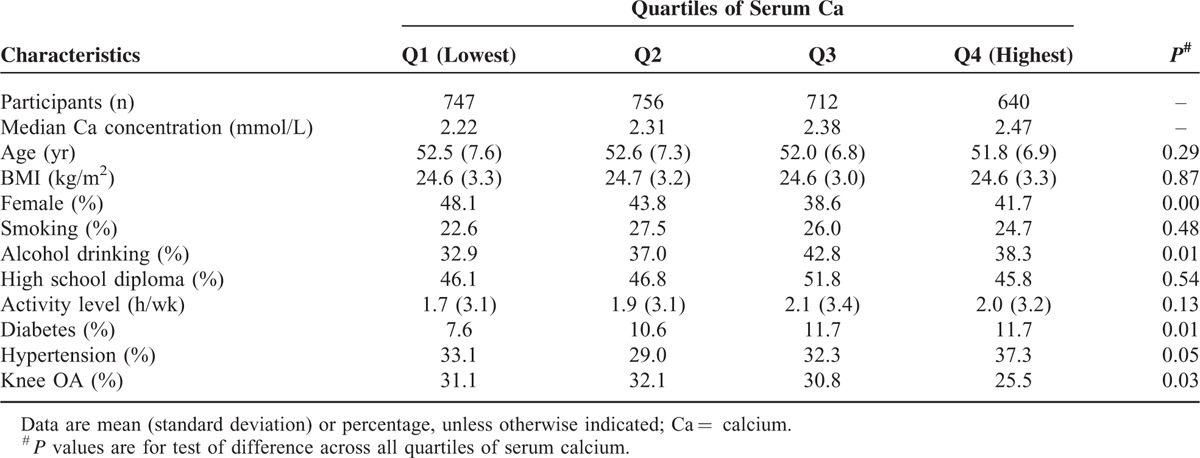
Characteristics Among 2855 Participants According to Quartiles of Serum Calcium

A negative association between serum calcium concentration and radiographic OA of the knee was observed in a model after adjustment for age, sex, and BMI, and also in a multivariable model adjusted for age, BMI, sex, educational level, smoking status, activity level, alcohol drinking status, diabetes, and hypertension (Table 2). The relative odds of radiographic OA of the knee were decreased by 0.79 times (OR = 0.79, 95% CI: 0.62–1.00) in the fourth quartile of serum calcium concentration compared with those in the lowest quartile after adjusted by age, sex, and BMI. A trend approached to be statistically significant (*P* = 0.07). The multivariable-adjusted ORs (95% CI) for radiographic OA of the knee from the first to the fourth serum calcium concentration quartiles were 1 (reference), 1.05 (95% CI: 0.83–1.31), 1.01 (95% CI: 0.80–1.27), and 0.79 (95% CI: 0.62–1.00) respectively, and a trend was also approached to be statistically significant (*P* = 0.06). The relative odds of radiographic OA of the knee were decreased by 0.79 times in the fourth quartile of serum calcium concentration compared with the lowest quartile. Subgroup analysis was also conducted to evaluate the association between serum calcium and radiographic OA in male and female population, respectively. The results suggested that the negative association still existed in the female population (multivariable adjusted OR = 0.66, 95% CI: 0.46–0.97 in the fourth quartile compared with the reference), but not in the male population.

**TABLE 2:**
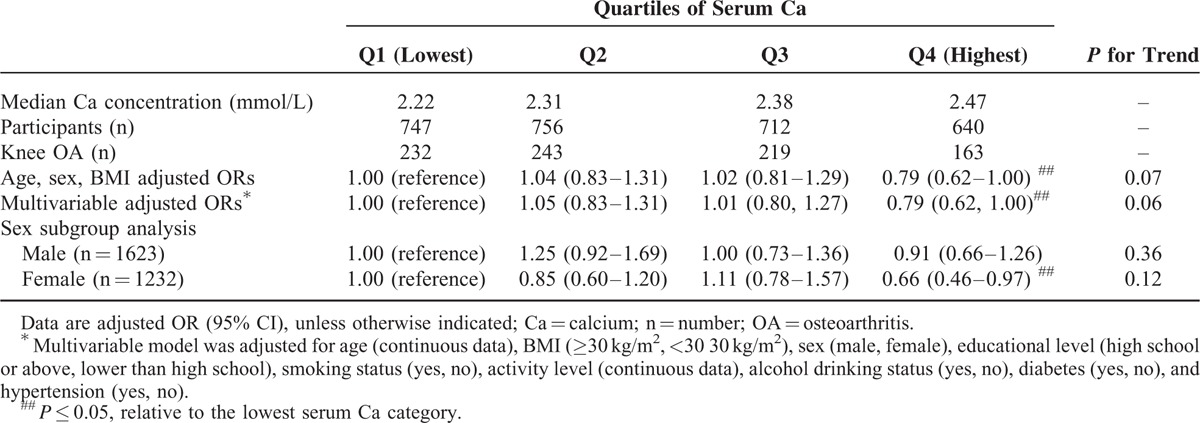
Multivariable-Adjusted Relations of Serum Ca and Radiographic Knee OA (n = 2855)

## DISCUSSION

In this population-based cross-sectional study, an inverse relationship between serum calcium and prevalence of radiographic OA of the knee was shown independent of some major confounding factors.

Yazmalar et al^19^ found that serum calcium levels were not significantly different between 74 knee osteoarthritis patients and 70 controls. The authors state that there were statistically significant differences between groups in terms of age, sex, and BMI (*P* < 0.05). However, these potential confounding factors were not adjusted, which may compromise the accuracy of the research results. In a case-control study conducted by Zoli et al,^20^ no statistically significant difference in calcium serum levels was found among hand osteoarthritis patients and healthy controls. Site difference may explain the different results observed. Hunter et al^21^ also failed to report an association between serum calcium and radiological features of knee OA in the cotwins with OA from 229 female white twin pairs aged from 24 to 79 years. Different from our study that measured serum calcium in patients with different K–L grades, this study compared serum calcium with radiological evidence of knee osteophytes with those without osteophytes, which may lead to the different results. What is more, the inconsistency between our study with previous study could also be due to difference of geographic setting. Moreover, previous studies are limited by a relatively small sample size.

The inverse relationship between serum calcium concentration and radiographic OA of the knee is biologically plausiable. In vitro studies have investigated the pathophysiological mechanism of calcium on chondrocyte. For chondrocytes, calcium is involved in matrix synthesis, cytoskeletal remodeling, cell hyperpolarization, and cell death. Intracellular calcium release has been implicated for aggrecan gene upregulation in response to static compression in cartilage explants.^27^ Cell experiment also has shown a requirement for calcium to complete post-translational modifications of glycosylation and export of secretory proteins.^28,29^ Amin's study suggested that extracellular calcium may be important for maintaining calcium homeostasis and cell viability over time.^30^ Even though there is no direct evidence found that calcium plays a role in the pathogenesis of OA, these studies support that calcium is involved in physiological and pathological processes of chondrocyte. Chondrocytes functions may be impaired under conditions of calcium insufficiency. Certainly, studies are still required to clearly understand the mechanisms of efficacy and action of calcium. In addition, significant differences were observed across all quartiles of serum calcium concentration in terms of female ratio, alcohol drinking ratio, the ratio of diabetes, and the ratio of hypertension. These findings were in accordance with some previous studies that indicated that serum calcium levels are associated with alcohol drinking,^31,32^ sex difference,^33,34^ diabetes,^35,36^ and hypertension,^37,38^ and need future studies to elaborate further.

It is well known that Mg and calcium antagonize each other in various physiological activities. And they may directly or indirectly compete for intestinal absorption. A high calcium intake consistently leads to significantly increased excretion of Mg via urine.^6,39,40^ It should be mentioned that serum Mg concentration may also have an inverse relationship with knee OA which was indicated by our previous study.^5^ However, to our best knowledge, there was no study reported that serum calcium level is negatively associated with serum Mg level. Thus, our findings may not contradict previous researches. In addition, the association between dietary Mg intake and serum Mg level may be influenced by some factors, such as renal function and diabetes.^41,42^ So maybe lower serum Mg level does not necessarily lead to increased calcium reabsorption. Furthermore, Nielsen et al reported that Mg deficiency-induced calcium retention most likely did not increase the amount of calcium as bone mineral and did not increase extracellular calcium, but instead increased soft tissue calcium concentrations.^43^ So Mg deficiency does not necessarily lead to increased serum calcium level. Overall, serum calcium and Mg levels are influenced by a lot of factors and the findings of our studies are worth exploring by further studies.

Our present study has several strengths. To the best of our knowledge, this is the first study that examined the association between serum calcium concentration and radiographic OA of the knee in a large sample in Asia. Second, the multivariable model was adjusted for a considerable number of potentially confounding factors, which greatly improves the reliability of the results.

Several limitations of this study must be taken into account when interpreting the results. First, because it was an observational study, a definitive causal relation could not be drawn. We anticipate that future prospective studies and intervention trials will help to clarify a causal association between serum calcium concentration and knee OA. Second, the serum level of parathyroid hormone (PTH) and vitamin D was not measured. Both PTH and vitamin D play a crucial role in calcium metabolism, so it is impossible to absolutely exclude confounding factors such as primary hyperparathyroidism and secondary hyperparathyroidism due to vitamin D deficiency. Third, there was no repeat calcium measurement during follow-up. A single measurement of plasma calcium levels could merely reflect a snapshot at a particular time, and long-term calcium status is not certain. We cannot comment on serum concentration before OA developed either. Fourth, we did not examine the association between serum calcium level and knee OA among participants younger than 40 years. However, middle-aged and older adults are considered at high risk of knee OA, and the prevalence of knee OA among participants younger than 40 years may be relatively low. Last, other factors (including pain and functional level) potentially related to serum calcium were not available for all the participants. Thus, we are unable to take these factors into account.

In conclusion, the present study found that serum calcium concentration has an inverse relationship with radiographic OA of the knee. High level of calcium may possibly exert a protective role in the control of radiographic knee OA.
